# Supplementary material for: Combined effects of water temperature, grazing snails and terrestrial herbivores on leaf decomposition in urban streams
Source: PeerJ. 2019 Oct 8;7:e7580. doi: 10.7717/peerj.7580 (PMC6788434; doi:10.7717/peerj.7580)
Supplement: Supplemental Information 1 [file peerj-07-7580-s006.docx]

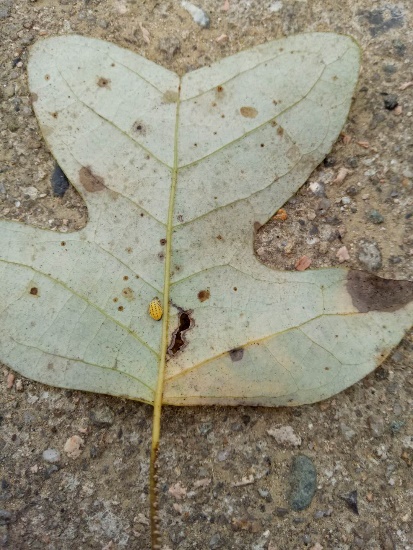

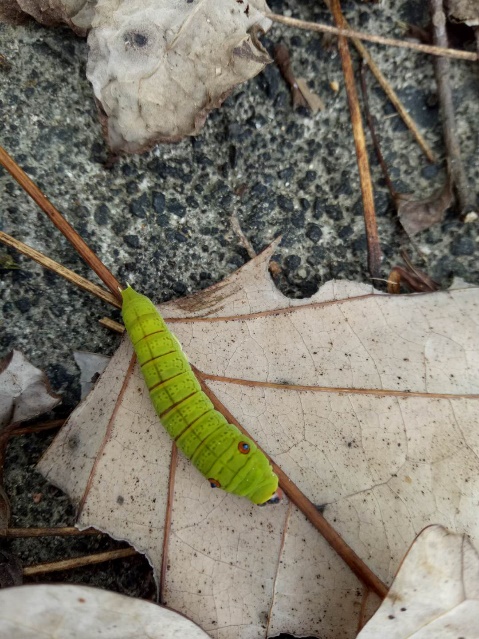

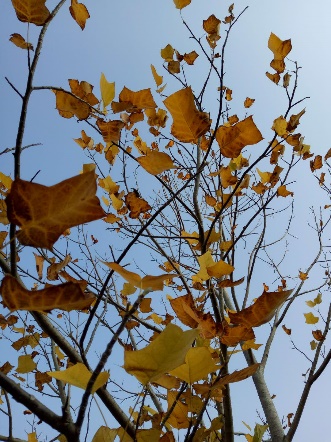


C

B

A

Figure S1 Terrestrial insects found on the leaves (A and B) and intact leaf litter (C).


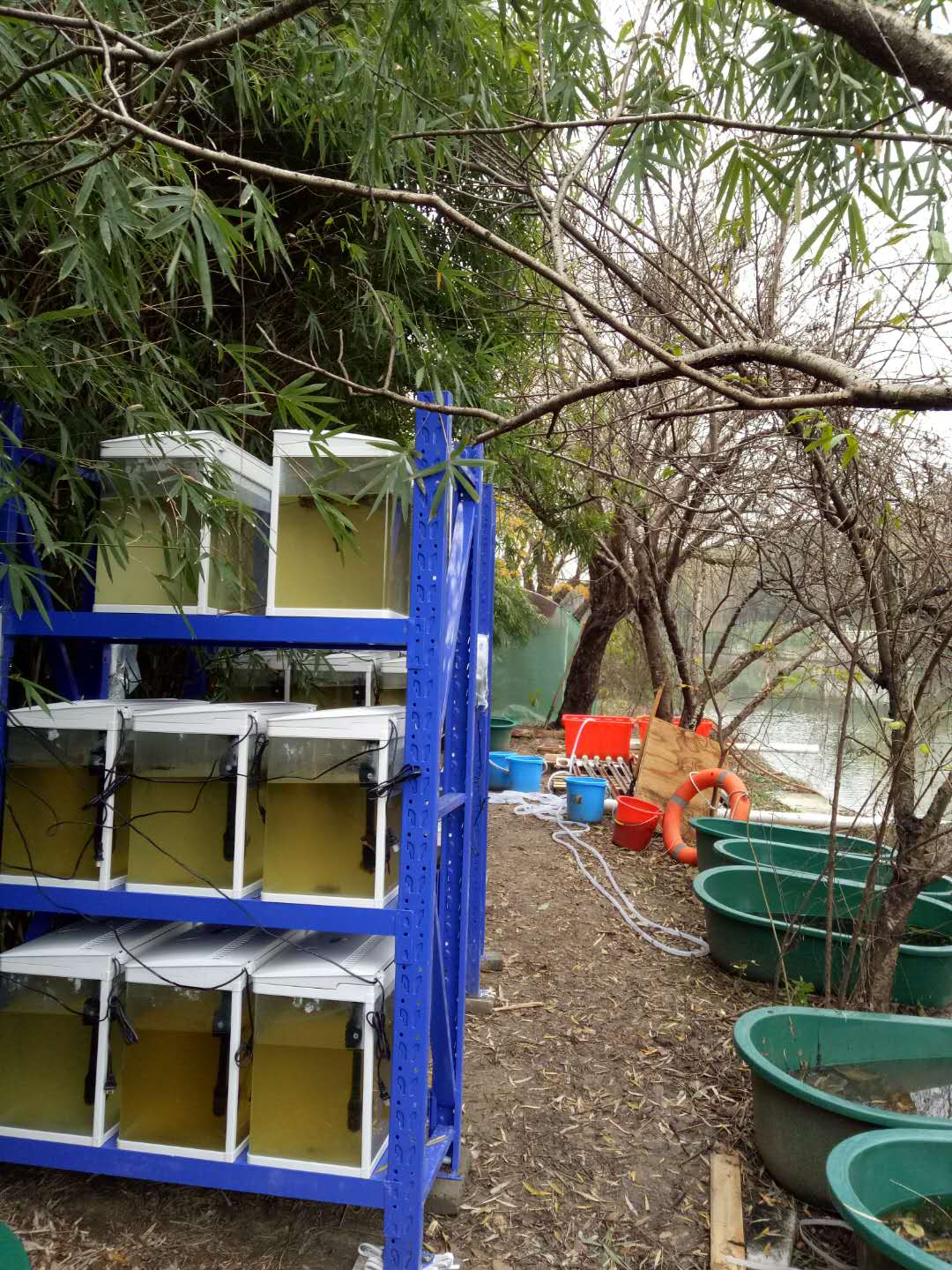


Figure S2 Experimental Mesocosms installed on the riparian zone of a stream.


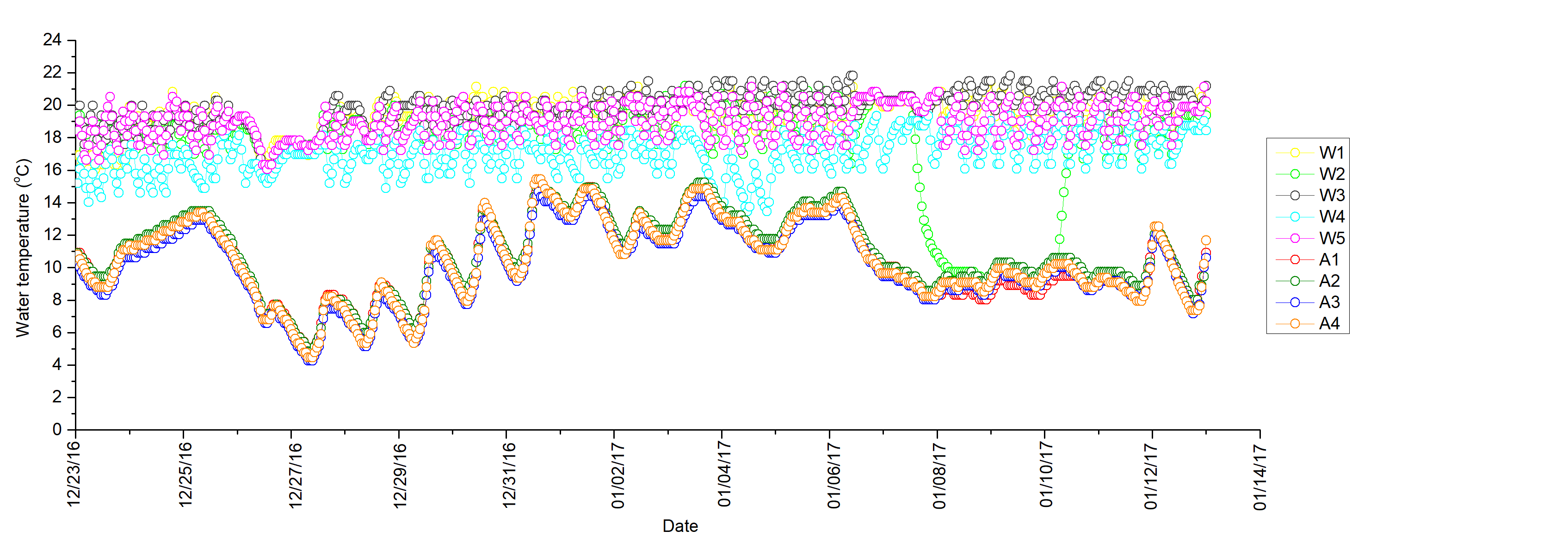


Figure S3 Hourly changes of water temperature in five warming (W1-W5) and four ambient (A1-A4) treatments during the experimental period.


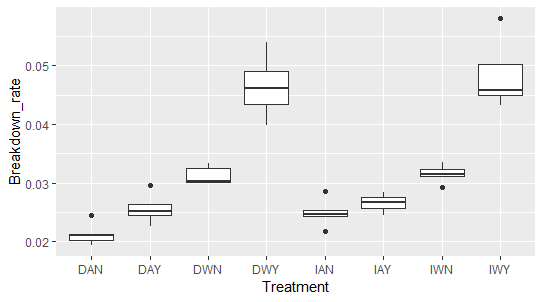


Figure S4 Boxplot of leaf litter breakdown rates for each treatment. Letters in the x axis mean: D (insect damaged); I (intact leaves); A (ambient temperature); W (warming); N (without snails); Y (with snails).


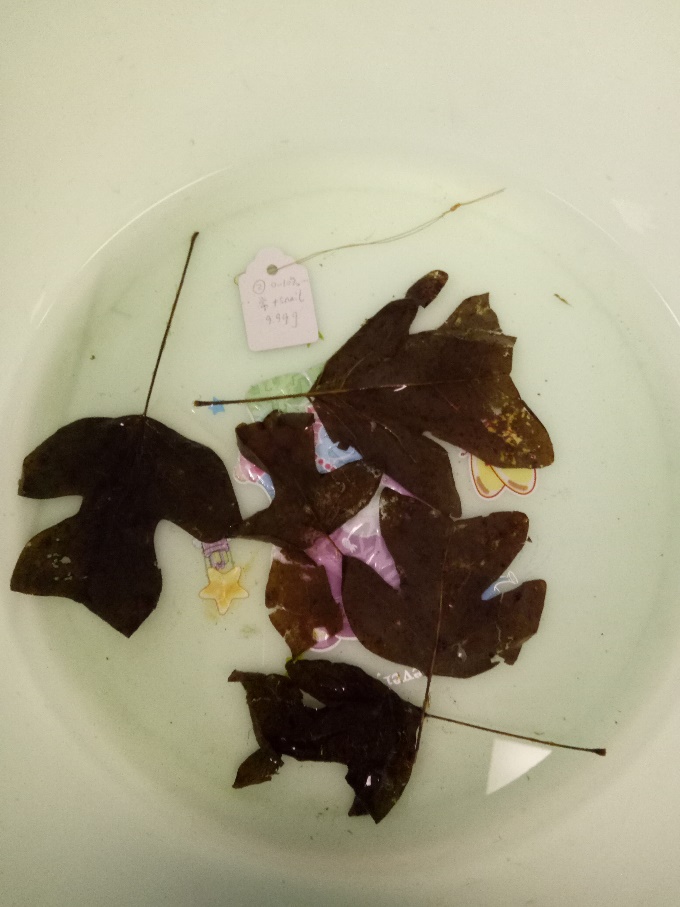

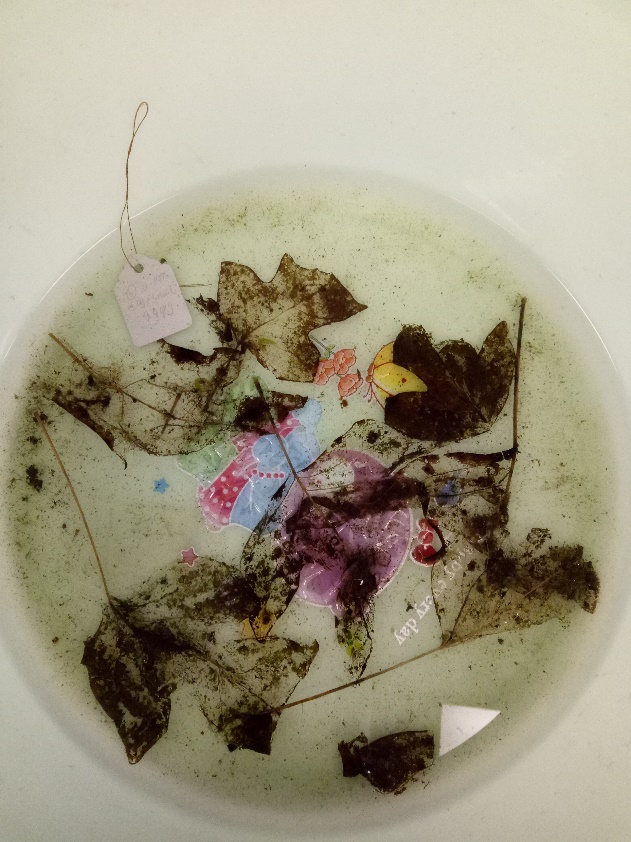


B

A


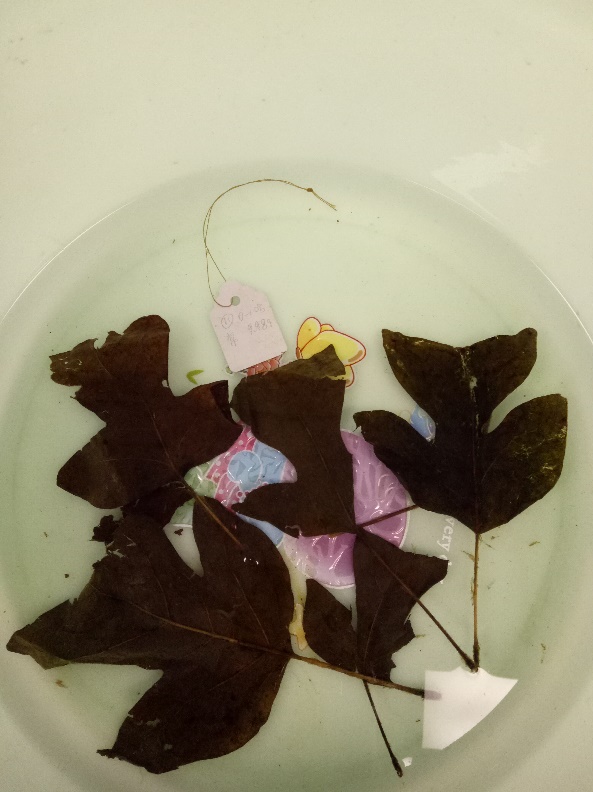

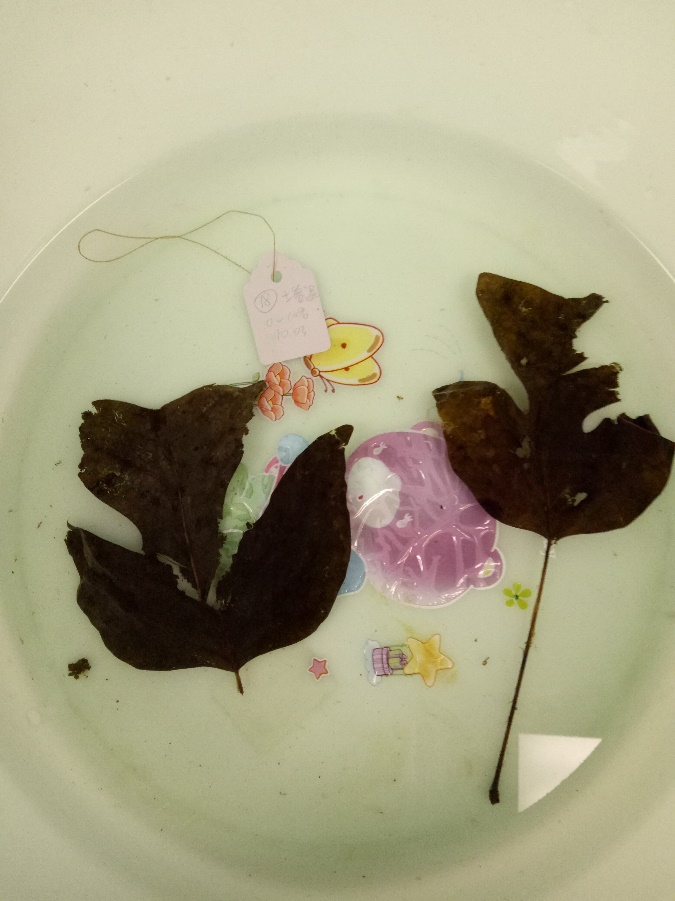


D

C


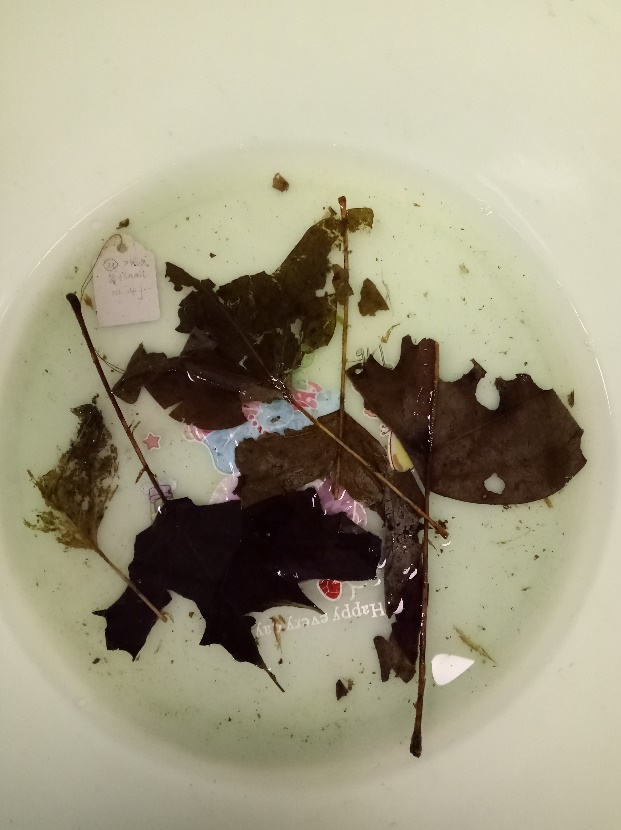

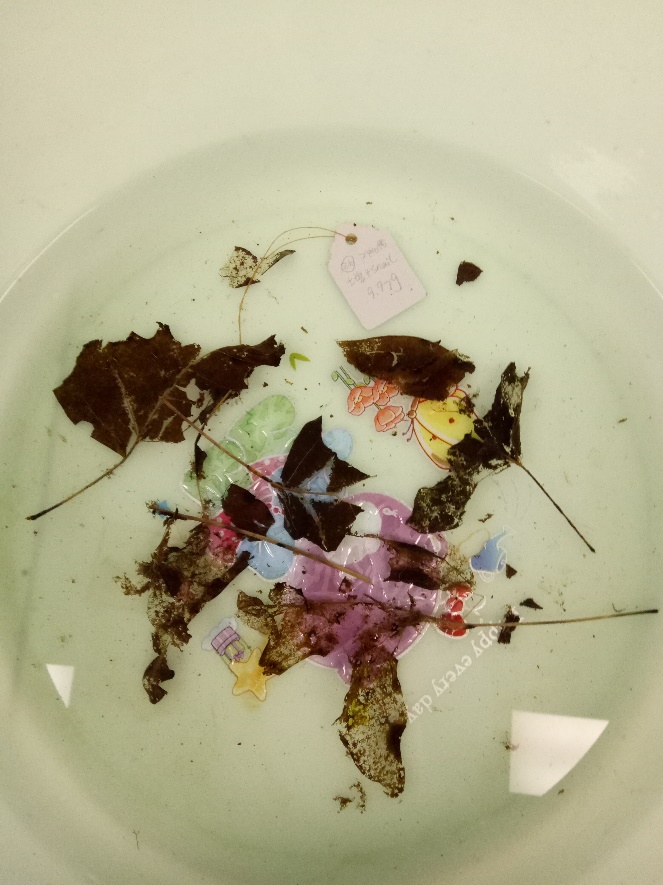


F

E


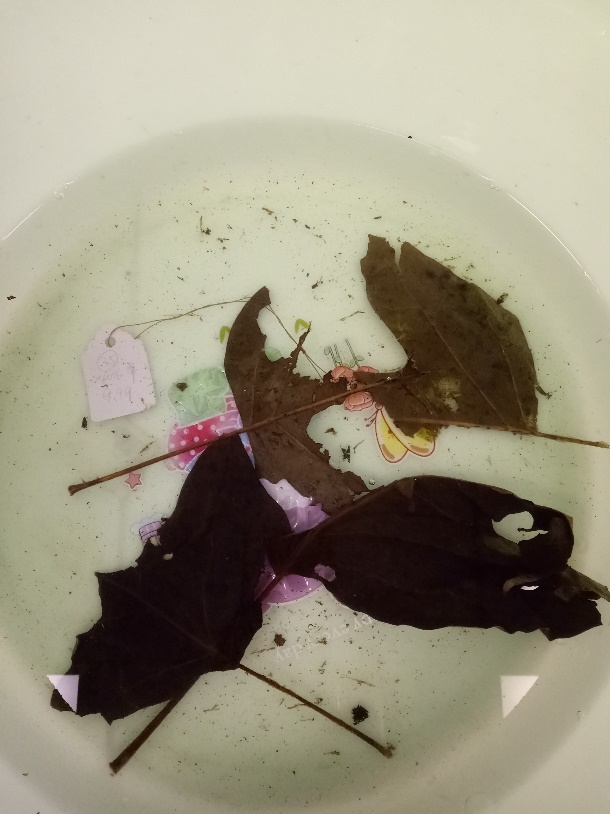

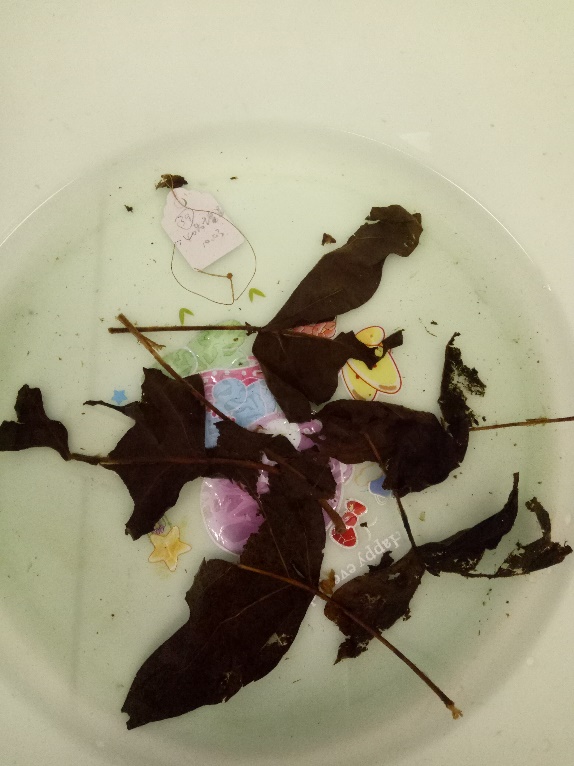


G

H

Figure S5 Leaf appearance after the experiment (Day 25) in treatments of (A) intact leaves + ambient temperature + snails; (B) intact leaves + warming temperature + snails; (C) intact leaves + ambient temperature + no snails; (D) intact leaves + warming temperature + no snails; (E) terrestrial insect damaged leaves + ambient temperature + snails; (F) terrestrial insect damaged leaves + warming temperature + snails; (G) terrestrial insect damaged leaves + ambient temperature + no snails; and (H) terrestrial insect damaged leaves + warming temperature + no snails.

Table S1 Summary results of three-way ANOVA with repeated measures for the effects of water temperature (T), snail (S), and litter quality (Q) on water quality in experimental mesocosms. *P*-values < 0.05 are in bold print.

| Source of variation |  | pH |  | Turbidity (NTU) | | DO (%) | | DO (mg/L) | | Ammonia (mg/L) | |  | Conductivity (μs/cm) | |
| --- | --- | --- | --- | --- | --- | --- | --- | --- | --- | --- | --- | --- | --- | --- |
| Between-Subjects effects | df | *F* | *P* | *F* | *P* | *F* | *P* | *F* | *P* | *F* | *P* | df | *F* | *P* |
| Q | 1 | 10.559 | **0.003** | 1.051 | 0.313 | 2.039 | 0.163 | 0.169 | 0.684 | 1.000 | 0.325 | 1 | 0.662 | 0.422 |
| T | 1 | 69.820 | **<0.001** | 424.048 | **<0.001** | 260.390 | **<0.001** | 513.690 | **<0.001** | 251.504 | **<0.001** | 1 | 300.323 | **<0.001** |
| S | 1 | 16.757 | **<0.001** | 51.027 | **<0.001** | 5.357 | **0.027** | 0.598 | 0.445 | 30.959 | **<0.001** | 1 | 0.878 | 0.356 |
| Q × T | 1 | 2.182 | 0.149 | 0.747 | 0.394 | 0.008 | 0.928 | 0.574 | 0.454 | 1.361 | 0.252 | 1 | 1.042 | 0.315 |
| Q × S | 1 | 21.549 | **<0.001** | 0.782 | 0.383 | 1.517 | 0.227 | 0.784 | 0.383 | 5.189 | **0.030** | 1 | 0.523 | 0.475 |
| T × S | 1 | 1.711 | 0.200 | 14.898 | **0.001** | 0.718 | 0.403 | 0.049 | 0.827 | 8.302 | **0.007** | 1 | 0.520 | 0.476 |
| Q × T × S | 1 | 1.603 | 0.215 | 10.346 | **0.003** | 3.021 | 0.092 | 1.512 | 0.228 | 0.151 | 0.700 | 1 | <0.001 | 0.996 |
| Error | 32 |  |  |  |  |  |  |  |  |  |  | 32 |  |  |
| Within-Subjects effects |  |  |  |  |  |  |  |  |  |  |  |  |  |  |
| Time | 2 | 44.196 | **<0.001** | 230.871 | **<0.001** | 59.741 | **<0.001** | 153.381 | **<0.001** | 195.765 | **<0.001** | 1.434 | 15.607 | **<0.001** |
| Time × Q | 2 | 1.842 | 0.167 | 3.653 | **0.031** | 1.173 | 0.316 | 0.359 | 0.700 | 6.153 | **0.004** | 1.434 | 4.101 | **0.035** |
| Time × T | 2 | 29.390 | **<0.001** | 14.033 | **<0.001** | 6.438 | **0.003** | 76.869 | **<0.001** | 3.181 | **0.048** | 1.434 | 93.201 | **<0.001** |
| Time × S | 2 | 0.635 | 0.533 | 10.012 | **<0.001** | 2.683 | 0.076 | 1.400 | 0.254 | 0.317 | 0.729 | 1.434 | 0.833 | 0.406 |
| Time × Q × T | 2 | 6.254 | **0.003** | 4.861 | **0.011** | 0.465 | 0.630 | 0.707 | 0.497 | 2.164 | 0.123 | 1.434 | 0.587 | 0.506 |
| Time × Q × S | 2 | 4.903 | **0.010** | 4.596 | **0.014** | 0.147 | 0.864 | 0.002 | 0.998 | 4.263 | **0.018** | 1.434 | 0.806 | 0.416 |
| Time × T × S | 2 | 0.204 | 0.816 | 0.136 | 0.873 | 0.310 | 0.735 | 0.573 | 0.567 | 0.900 | 0.411 | 1.434 | 0.399 | 0.605 |
| Time × Q × T × S | 2 | 1.601 | 0.210 | 5.110 | **0.009** | 2.554 | 0.086 | 2.549 | 0.086 | 1.270 | 0.288 | 1.434 | 0.570 | 0.514 |
| Error | 64 |  |  |  |  |  |  |  |  |  |  | 45.895 |  |  |
