# Supplementary material for: Combined effects of water temperature, grazing snails and terrestrial herbivores on leaf decomposition in urban streams
Source: PeerJ. 2019 Oct 8;7:e7580. doi: 10.7717/peerj.7580 (PMC6788434; doi:10.7717/peerj.7580)
Supplement: Supplemental Information 2 — Background water quality during the experimental period. [file peerj-07-7580-s007.docx]

**Supplementary methods**

***Water quality*** Warming significantly affected all measured water quality variables (Table 1 and Table S1 and Dataset S2 in supporting information, Fig. 2), which was also found in other studies (Martínez et al. 2014, Domingos et al. 2015, Ferreira et al. 2015). Both pH and conductivity increased with increasing water temperature, while turbidity, dissolved oxygen (DO), and ammonia were reduced in warming treatments. The presence of snails decreased pH, turbidity, and DO. By contrast, snails increased ammonia and had no significant effect on conductivity. Litter quality only significantly affected pH by increasing pH in mesocosms containing insect-damaged litter. Most two-way interactions showed additive effects on water quality variables and only turbidity was affected by the three-way interaction.
